# A Binocular Stereo-Imaging-Perception System with a Wide Field-of-View and Infrared- and Visible Light-Dual-Band Fusion

**DOI:** 10.3390/s24020676

**Published:** 2024-01-17

**Authors:** Youpan Zhu, Dan Zhang, Yongkang Zhou, Weiqi Jin, Lingling Zhou, Guanlin Wu, Yong Li

**Affiliations:** 1 MOE Key Laboratory of Optoelectronic Imaging Technology and System, Beijing Institute of Technology, Beijing 100081, China; zhuypbit@aliyun.com (Y.Z.); 3120220595@bit.edu.cn (G.W.); 2 Kunming Institute of Physics, No. 31, Jiaochang East Road, Wuhua District, Kunming 650223, China; zyk1120102464@163.com; 3 Yunnan North Optical & Electronic Instrument Co., Ltd., No. 300, Haikou Town, Xishan District, Kunming 650114, China; zhangdanynoei@163.com (D.Z.); lynzhll@163.com (L.Z.); 4 School of Life Science, Beijing Institute of Technology, 5 South Zhongguancun Street, Haidian District, Beijing 100081, China; 5 Chengdu Zhongke Information Technology Co., Ltd., No. 1369 Kezhi Road, Xinglong Street, Tianfu New District, Chengdu 610042, China; scly2012@163.com

**Keywords:** binocular stereoscopic-perception system, wide field-of-view, infrared and visible light fusion, image distortion

## Abstract

With the continuous evolution of autonomous driving and unmanned driving systems, traditional limitations such as a limited field-of-view, poor ranging accuracy, and real-time display are becoming inadequate to satisfy the requirements of binocular stereo-perception systems. Firstly, we designed a binocular stereo-imaging-perception system with a wide-field-of-view and infrared- and visible light-dual-band fusion. Secondly we proposed a binocular stereo-perception optical imaging system with a wide field-of-view of 120.3°, which solves the small field-of-view of current binocular stereo-perception systems. Thirdly, For image aberration caused by the wide-field-of-view system design, we propose an ellipsoidal-image-aberration algorithm with a low consumption of memory resources and no loss of field-of-view. This algorithm simultaneously solves visible light and infrared images with an aberration rate of 45% and 47%, respectively. Fourthly, a multi-scale infrared- and visible light-image-fusion algorithm is used, which improves the situational-awareness capabilities of a binocular stereo-sensing system in a scene and enhances image details to improve ranging accuracy. Furthermore, this paper is based on the Taylor model-calibration binocular stereo-sensing system of internal and external parameters for limit correction; the implemented algorithms are integrated into an NVIDIA Jetson TX2 + FPGA hardware framework, enabling near-distance ranging experiments. The fusion-ranging accuracy within 20 m achieved an error of 0.02 m, outperforming both visible light- and infrared-ranging methods. It generates the fusion-ranging-image output with a minimal delay of only 22.31 ms at a frame rate of 50 Hz.

## 1. Introduction

In the field of autonomous driving, the primary methods for perception systems currently include millimeter-wave radar perception, LiDAR perception, millimeter-wave radar, and LiDAR fusion with visual perception, monocular vision, and binocular stereo vision. Radar- and vision sensor-fusion methods involve the calibration of multiple sensors in both time and space, transforming their acquired data into a common coordinate system, followed by information processing. The authors in [1] used the radar sensor and visual sensor approaches to investigate the vehicle environment. This approach is more accurate in terms of the information obtained but it suffers from the complexity of calibrating multiple sensors with inconsistent sensing ranges and becomes increasingly expensive as accuracy increases. Monocular stereo vision utilizes a single camera to project the three-dimensional world onto a two-dimensional plane, resulting in a loss of depth information. Monocular stereo vision systems assume static ground and solve for a dynamic camera pose to achieve distance-measurement functionality [2,3,4]. R. Mur-Artal et al. [5] introduced the ORB-SLAM monocular simultaneous localization and mapping (SLAM) system, which exhibits robustness against significant motion noise and allows for wide baseline-loop closure and relocalization but requires further improvements in depth accuracy. Raul Mur-Artal et al. [6] presented ORB-SLAM2, a monocular SLAM system that employs bundle adjustment to enhance depth accuracy and features a lightweight localization mode for the efficient reutilization of disabled maps. Carlos Campos et al. [7] introduced ORB-SLAM3, which employs comprehensive short-term, medium-term, and long-term data associations to achieve zero drift in mapped areas. Compared to other methods, monocular stereo vision systems offer the advantage of being small and lightweight. However, such systems can only capture two-dimensional images and cannot determine distances to objects, leading to issues with low ranging accuracy and poor environmental adaptability. Binocular stereo vision, on the other hand, emulates the human visual system and leverages the principle of disparity to acquire target features from different positions using detectors. This approach establishes the relationship between corresponding pixel points in the image based on the three-dimensional geometric positions of certain features on the spatial-target surface. This process involves constructing a binocular stereo vision-imaging model to obtain model parameters [8,9]. Binocular stereo vision is also referred to as passive binocular depth sensing and offers improved robustness in depth computation compared to monocular stereo vision. This technology also enables three-dimensional object recognition. Various methods exist for obtaining real-world depth information in binocular stereo perception systems, such as passive stereo [10], active stereo [11], time-of-flight imaging [12], and defocus depth [13]. In this paper, the passive stereo method of binocular stereo systems is employed.

With the advancement of industrial automation and machine vision, binocular stereo perception systems have been widely researched and applied in various fields, including vehicle driving [14], intelligent transportation [15], 3D reconstruction [16], virtual reality [17], surveying, and rapid positioning [18]. In the realm of autonomous and assisted driving [19], the first assisted-driving vehicle based on a binocular stereo vision system was developed in Japan. This system processes images captured by two onboard cameras. Subsequently, in Europe and the United States, autonomous driving vehicles were developed based on binocular stereo vision. Notable examples include the NavLab series developed at Carnegie Mellon University in the United States [20] (NavLab-1, NavLab-5, and NavLab-11). These vehicles utilize binocular stereo vision for road environment detection and focus on addressing challenging visual perception issues in complex environments. Silicon Valley chip company Ambarella has developed binocular ADAS (Advanced Driver Assistance Systems) and autonomous driving chips [21], as well as binocular stereo-specific chips and solutions tailored to binocular vision systems, which act as valuable complements to integrated chips. These specialized chips can handle a portion of the perception tasks at the edge, creating a smaller and more efficient perception-decision loop.

With the evolution of deep learning, many researchers have proposed more efficient and accurate binocular stereo algorithms. Sun et al. [22] introduced a multi-path Viterbi (MPV) multi-scale fast stereo matching algorithm for generating dense disparity information. Jure Bontar and others [23] trained a convolutional neural network (CNN) to predict the matching degree between two image patches and used this CNN for binocular stereo matching. The authors incorporated left–right consistency checks to eliminate errors in occluded areas. However, their method was found to be ineffective in textureless regions. Li et al. [24] introduced Stereo R-CNN, which takes binocular images as network inputs. The authors extended Faster R-CNN to simultaneously detect and associate objects in left and right images. Through the binocular FPN network, the authors predicted object key points and sizes to generate coarse 3D object detection boxes. Finally, the authors used the region-based photometric correspondence method to refine fine-grained 3D detection boxes. Tang et al. [25] used the YOLOv4-tiny model to generate boundary frameworks and employed an adaptive stereo matching approach based on these boundaries. This approach meets real-time detection requirements and exhibits high localization stability and robustness under varying lighting conditions.

Autonomous driving vehicle is a kind of autonomous and automatic unmanned vehicle, the vehicle not only needs to grasp the dynamic situation of nearby vehicles and obstacles in real-time, but also needs to deal with the complex environment of night, haze, and obstacles. The traditional driving system based on visible light is easy to fail in the area of poor lighting conditions, unable to meet the all-weather needs of automatic driving vehicles, infrared images are not subject to the interference of illumination, sun glare, can penetrate smoke, haze, and other characteristics. The perception ability of automatic driving vehicles can be further improved by fusion of visible light and infrared. Current autopilots commonly use small field-of-view cameras, usually with a field-of-view of 40–60, which limits the system’s perception of the external environment, and the field-of-view and perception can be further improved to a wide-field-of-view technology with infrared- and visible light fusion [26].

The field of autonomous driving places significant emphasis on important metrics such as the field-of-view, ranging accuracy, and real-time display in binocular stereo perception systems. This paper integrates the characteristics of visible light and infrared sensors to design a wide-field-of-view binocular stereo imaging perception system using infrared- and visible light-fusion . This system combines a wide-field-of-view binocular stereo optical imaging system with a low-storage image distortion correction algorithm to achieve imaging with a wide field-of-view and minimal distortion. Focusing on the features of visible light and infrared images, this study employs a multi-scale fusion approach for infrared- and visible light images. This work explores a straightforward yet precise method for object ranging based on the Taylor model for calibrating the internal and external parameters of the binocular stereo perception system for extreme correction, thereby enhancing the measurement accuracy of the binocular stereo perception system. The algorithms are implemented using the NVIDIA Jetson TX2 + FPGA hardware framework, enabling real-time ranging and display.

## 2. System Framework

**Figure 1:**
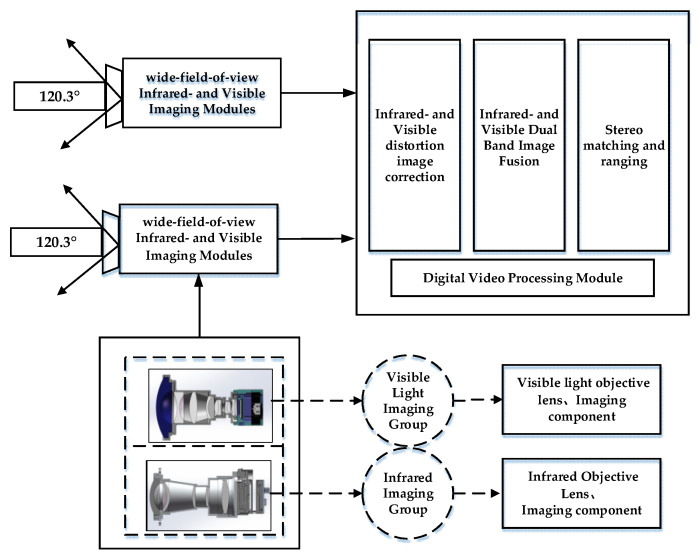
Schematic diagram of the wide-field binocular stereo perception system with both infrared- and visible light components.

**Figure 2:**
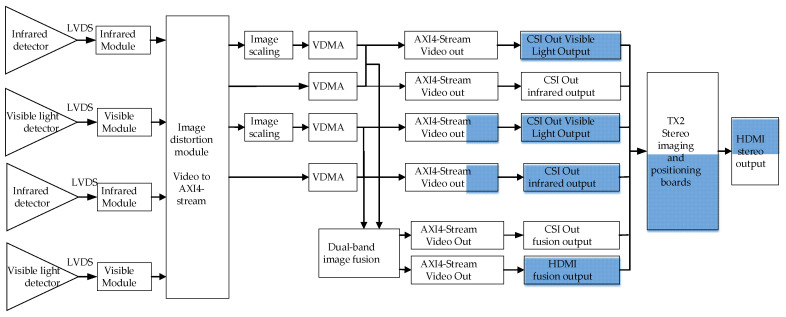
The digital-video-image-processing module.

In this paper, a wide-field-of-view binocular stereo sensing system based on infrared and visible light is established; the system block diagram is shown in Figure 1. This system consists of two sets of uncooled long-wave infrared-imaging modules with a field-of-view greater than 120°, visible-high-definition-imaging modules, and digital-video-processing modules. The system’s operating temperature ranges from −40 °C to 70 °C. The digital-video-processing module utilizes hardware circuits featuring the NVIDIA Jetson TX2 + FPGA combination and receives digital video signals from the visible light-imaging component and the infrared-imaging component, enabling the module to perform image processing. A hardware diagram of the digital-video-processing module is shown in Figure 2.

### 2.1. Design and Simulation of the Wide-Field Binocular Stereo Perception-Dual-Band-Imaging System

#### 2.1.1. The Principle of Binocular Stereo Imaging

In response to the specific requirements of night-time vehicle driving and rapidly changing work environments, we designed a wide-field-binocular stereo perception system with dual-band-electro-optical imaging. The visiblelight-imaging component incorporates a large-array CMOS device from Rockchip Electronics Co., Ltd. (Jiangsu, China) that has high sensitivity and definition. This system boasts a pixel resolution of 1920 (H) × 1080 (V), with each pixel measuring 13 μm (H) × 13 μm (V), and it operates at a frame rate of 50 Hz. This component can function effectively under low-light conditions down to 10^−3^ lx. Furthermore, the infrared-imaging component employs a non-cooled infrared focal plane detector from Yantai IRay Technology Co., Ltd. (Yantai, China) that offers a pixel resolution of 1024 (H) × 768 (V), with each pixel measuring 14 μm. The frame rate of the infrared component is also 50 Hz.

**Figure 3:**
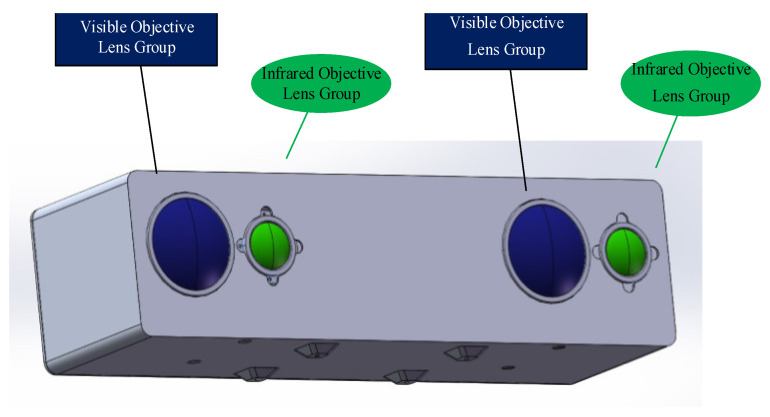
Schematic diagram of the infrared- and visible light-lens structure in the binocular-stereo-perception system.

Binocular stereo sensing systems are mainly divided into two types according to their placement [27]: a parallel model and a convergent model. The parallel model has two camera optical axes parallel to each other. Moreover, the structure is simple and easy to calculate. The advantage of this structure is the presence of only a negative horizontal parallax, with no vertical parallax. Disadvantages include a small common area and a lack of stereoscopic information in the left and right sides of the single viewing area, which will cause a waste of information. The convergence model can adjust the angle between the two optical axes to obtain a larger effective field-of-view, with positive-, negative-, or zero-horizontal parallax; however, the camera body will produce vertical parallax, which causes a certain gradient distortion. In this study, considering the advantages and disadvantages of the parallel model and the convergence model, the visible and infrared components were fused. For this purpose, we selected an optical design based on the parallel model, as shown in Figure 3. To realize binocular stereo vision, the binocular stereo sensing system consisted of two visible light-objective lens groups and two infrared-objective lens groups.

**Figure 4:**
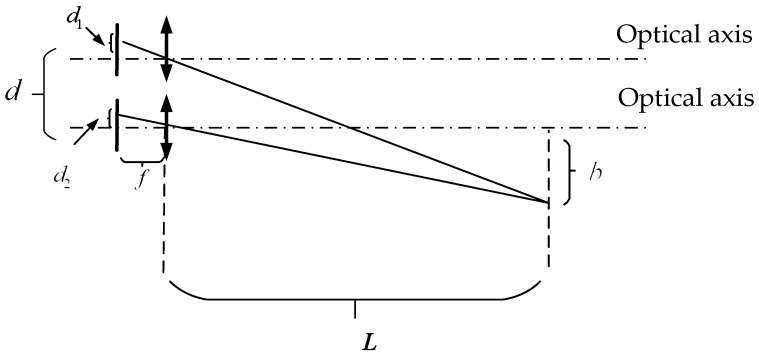
The parallel dual-axis-ranging model with the same focal length.

Binocular-imaging-distance-measurement technology relies on the binocular disparity to establish an ideal model for binocular ranging. In this model, both cameras have identical specifications and parameters, including matching camera models, consistent focal lengths, and the parallel alignment of optical axes. The model is shown in Figure 4.

Here, d represents the camera’s inter-image plane spacing (baseline width), $d_{1}$ and $d_{2}$ are the distance from the image point to the image plane center, f is the camera’s focal length, and L is distance to the object. Using the principles of similar triangles, the following relationships can be derived:

(1) $$L=\frac{fd}{d_{1}-d_{2}}=\frac{fd}{c}$$

where c represents the difference in the imaging positions of the object point in the two fields of view, which is commonly referred to as the parallax value.

Under the conditions of parameter determination in a binocular-stereo-perception system (such as focal length and baseline), ranging accuracy is determined by the parameter c . Presently, binocular stereo-matching algorithms can achieve sub-pixel-level matching accuracy, resulting in superior disparity precision. Results can be obtained by differentiating Equation (1):

(2) $$dL=\frac{fd}{c^{2}}dc=\frac{L^{2}}{fd}dc.$$

Hence, as long as the parameters of the binocular-stereo-perception system are determined, the distance to the target can be calculated by measuring the disparity. System parameters typically consist of intrinsic and extrinsic parameters. Intrinsic parameters include the focal length, principal point coordinates, and distortion coefficients of the left and right cameras, among others. Extrinsic parameters encompass the relative transformation between the left and right cameras, involving rotation and translation matrices. Due to potential errors during camera installation, such as non-parallel alignment of the lens and imaging plane, it is necessary to recalibrate the camera to obtain updated focal lengths, intrinsic parameters, and extrinsic parameters.

#### 2.1.2. The Simulation and Design of Visible- and Infrared Objective Lenses

A. Simulation design for the visible light objective lens

**Figure 5:**
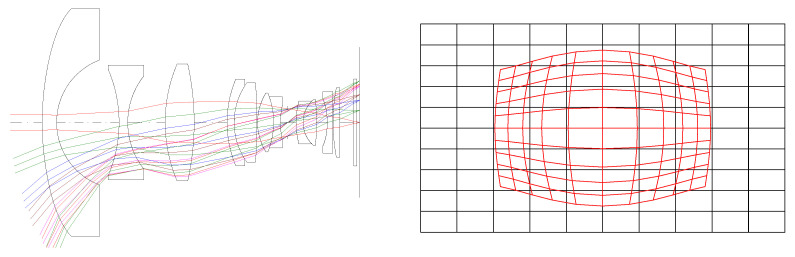
Visible light objective lens-optical system and distortion diagram.

In the wide-field binocular stereo perception-optical-imaging system, the visible light component employs a high-resolution and low-light CMOS-imaging module from Rockchip Electronics Co., Ltd., which enables imaging in both day and night scenes. This component features large pixels and sensor-imaging areas, imposing stringent requirements on the optical system. We utilized the CODE V(10.2) software for optical system design, implementing a “telephoto-type” optical path structure with 12 lenses. By introducing appropriate non-spherical elements while keeping the total number of lenses, glass thickness, and imaging quality constant, we enhanced the light-gathering capabilities of the visible light objective lens without compromising its transmittance. The system design is depicted in Figure 5. Table 1 presents the optical-design specifications for the visible light objective lens.

The maximum effective range L of the visible light-optical-imaging system is as follows:

(3) $$L=\frac{f\sqrt{wh}}{8d_{pix}}=97.16\;m$$

where w represents human height, h represents shoulder width, and $d_{pix}$ represents pixel size.

At a distance of L=15 m , there is a depth-calculation deviation of dL≤20 cm . Under the condition of a matching algorithm precision at the 0.1 pixel level, the baseline d can be determined as follows:

(4) $$d\geq \frac{L^{2}dC}{fdL}=13.52\;\mathrm{cm}$$

where L represents the distance from the target to the system, which is referred to as the test distance. dC is set at a 0.1 pixel level, and dL stands for the resolvable distance.

At a distance of L=30 m , the depth-calculation deviation of dL≤1 m . When these values are incorporated into Equation (4), the following result is obtained:

(5) $$d\geq \frac{L^{2}dC}{fdL}=10.82\;\mathrm{cm}.$$

It can be seen clearly that d≥13.52 cm . The formula for visible light stereo acuity dγ is

(6) $$d\gamma =\frac{\alpha d}{L^{2}}dL$$

where α is a constant of 206,265 when converting from radians to arcseconds:

(7) $$d\gamma =\frac{\alpha }{f}dc=24.47^{\prime \prime }.$$

Stereopsis is the ability to resolve the smallest horizontal disparity between retinal images from both eyes. The normal value for stereopsis should be less than 60″ . A smaller value of stereopsis indicates better stereo vision.

**Figure 6:**
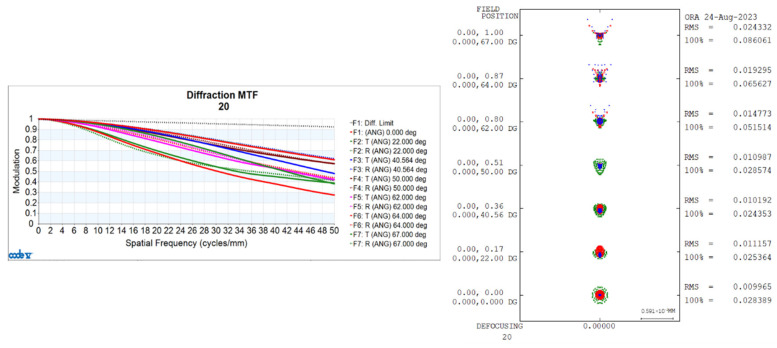
Transfer functions and diffuse spots of visible light-optical systems at 20 °C.

**Figure 7:**
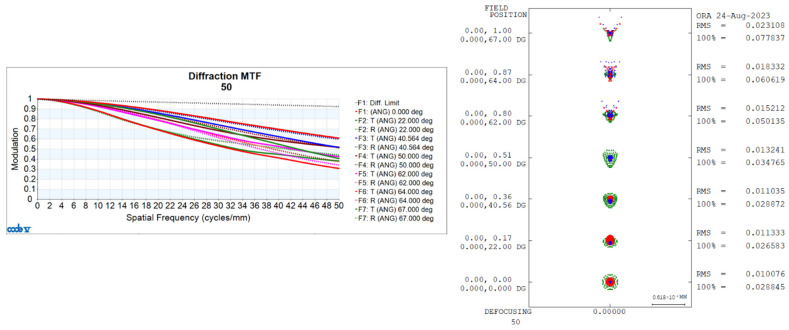
Transfer functions and diffuse spots of visible light-optical systems at 50 °C.

**Figure 8:**
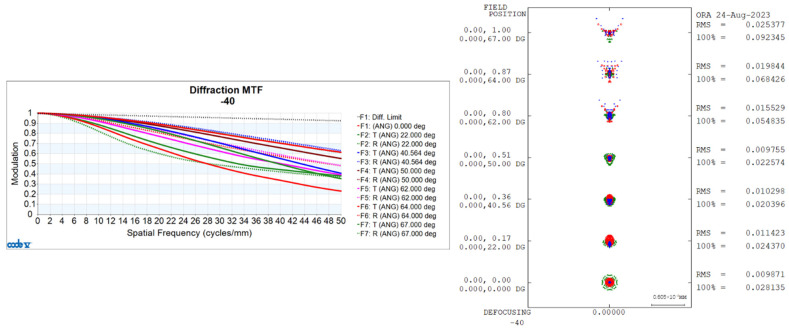
Transfer Functions and Diffuse Spots of Visible Light-Optical Systems at −40 °C.

Utilizing CONE V software, we simulated environmental temperature variations and obtained the transfer functions for the visible light-optical system at 20 °C, 50 °C, and −40 °C, as well as the diffuse spots, as shown in Figure 6, Figure 7, and Figure 8, respectively.

B. The Simulation Design of an Infrared objective lens

**Figure 9:**
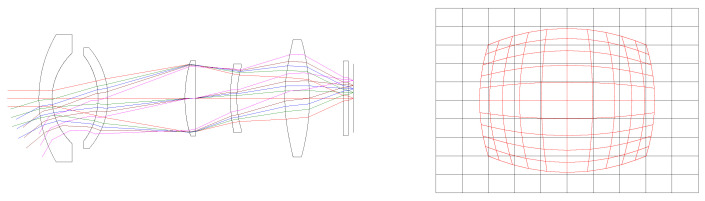
The optical system of the infrared objective lens and a distortion map of the infrared objective lens at 20 °C.

This paper addresses the design of a wide-field, relative aperture long-wave infrared optical system for the 1024 (H) × 768 (V) long-wave infrared detector from Yantai IRay Technology Co., Ltd. The infrared objective lens system, as depicted in Figure 9, operates without active cooling over a wide temperature range. The front surface of the first lens is non-spherical, enabling passive temperature compensation at different temperatures by adjusting the system’s back focal length. Table 2 provides the optical-design specifications for the infrared objective lens.

The maximum effective range L of the infrared objective lens-optical system is

(8) $$L=\frac{f\sqrt{wh}}{8d_{pix}}=40.67\;m.$$

At a distance of L=15 m , with a depth-calculation deviation dL≤30 cm , the baseline d can be calculated as follows:

(9) $$d\geq \frac{L^{2}dC}{fdL}=21.26\;\mathrm{cm}.$$

At a distance of L=30 m , with a depth-calculation deviation dL≤2 m , the baseline d can be calculated as follows:

(10) $$d\geq \frac{L^{2}dC}{fdL}=12.75\;\mathrm{cm}.$$

According to Formulas (9) and (10),

(11) d≥21.26 cm.

The formula for the stereo acuity dγ is as follows:

(12) $$d\gamma =\frac{\alpha d}{L^{2}}dL$$

where d is the baseline length, dL is the resolvable distance, L is the distance from the target to the system, and dL is the binocular ranging accuracy. The formula is as follows:

(13) $$d\gamma =\frac{a}{f}dc=58.44^{\prime \prime }$$

**Figure 10:**
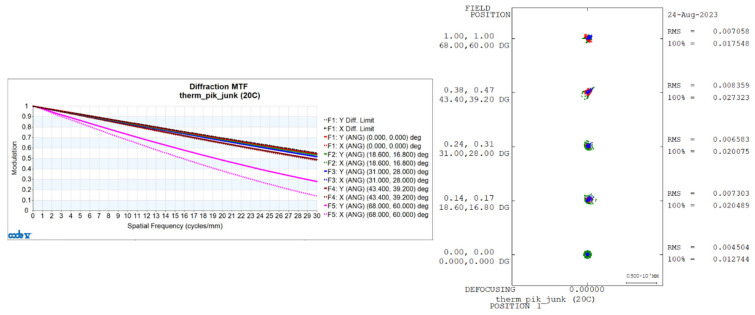
Transfer Functions and Diffuse Spots of Infrared Optical Systems at 20 °C.

**Figure 11:**
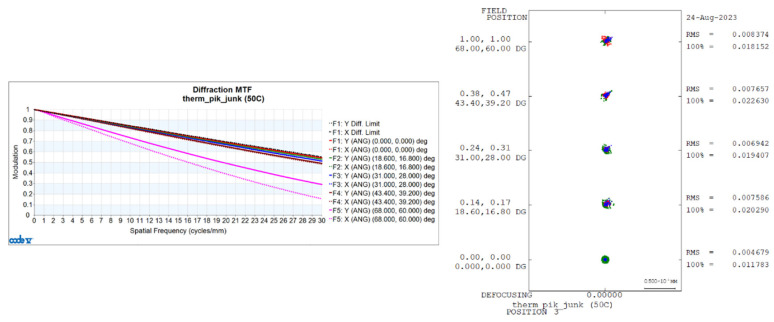
Transfer Functions and Diffuse Spots of Infrared Optical Systems at 50 °C.

**Figure 12:**
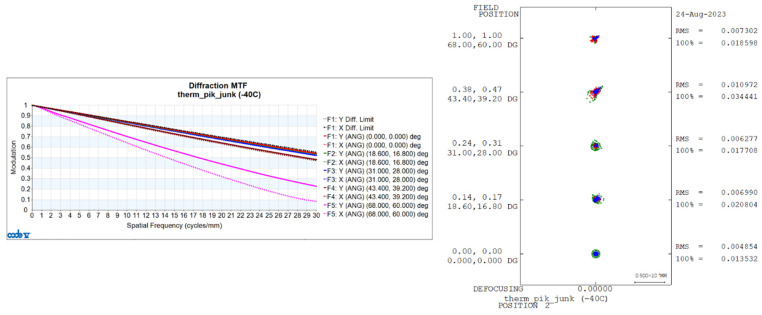
Transfer Functions and Diffuse Spots of Infrared Optical Systems at −40 °C.

With a decrease in temperature, the infrared optical system experiences changes in inter-lens spacing, lens thickness, refractive index, and curvature radius. Therefore, temperature variations inevitably lead to defocusing of the system’s focal plane, resulting in a degradation of image quality. The transfer functions and diffuse spots of the infrared optical system based on system simulation analysis are shown in Figure 10, Figure 11, and Figure 12, respectively, at temperatures of 20 °C, 50 °C, and −40 °C.

In summary, without moving any optical elements, we achieved alignment of the image plane with the detector target surface during changes in environmental temperature. With temperature variations, the lens assembly, employing optical passive thermal compensation, can maintain consistent magnification and requires no active optical components. The relative positions of the optical axes remain relatively unchanged, resulting in high image registration accuracy. Large-field optical systems often exhibit significant distortion to improve the field-of-view. Image distortion is corrected to obtain high-quality fused images. Based on the imaging quality of the fused lens system and image registration effectiveness, further improvements in the system’s ranging accuracy were achieved.

Baseline distance d≥21.26cm ; if baseline distance d=35cm , then the ranging accuracy is as follows:

(14) $$dL\geq \frac{L^{2}dC}{fd}.$$

Table 3 presents the calculated accuracy of visible and infrared ranging at test distances of 15 m and 30 m.

### 2.2. Real-Time Image Distortion Correction and Simulation

The optical system design model produces aberrations that are unavoidable due to the system’s non-coaxial nature, field-of-view, focal length, and other auxiliary factors. In this study, a binocular stereo sensing optical imaging system with a large field-of-view is designed, in which the aberration rate produced by the visible objective lens is −45%, and the aberration rate of the infrared objective lens is −47%, as shown in Section 2.1. Here, we propose a shareable elliptical aberration for real-time correction.

Image distortion is mainly the result of geometric distortion of the pixel positions of the image after imaging. Geometric distortion is further categorized into linear and nonlinear distortion, which refer to a mixture of several distortions that work together [28]. The causes of nonlinear aberrations are mainly categorized as radial aberrations, centrifugal aberrations, and thin prismatic aberrations [29,30]:

(15) $$x_{d}=x(1+k_{1}r^{2}+k_{2}r^{4}+k_{3}r^{6})+2p_{1}xy+p_{2}(r^{2}+2x^{2})$$

(16) $$y_{d}=y(1+k_{1}r^{2}+k_{2}r^{4}+k_{3}r^{6})+2p_{1}xy+p_{2}(r^{2}+2y^{2})$$

(17) $$x^{2}+y^{2}=r^{2}$$

where x and $x_{d}$ represent, respectively, the reference image and distortion image in the Xdirection; y and $y_{d}$ are, respectively, the reference image and distortion image in the Y-direction; $k_{1}$ , $k_{2}$ , $k_{3}$ , $p_{1}$ , and $p_{2}$ are distortion correction parameters. When the aberration coefficient is greater, the aberration correction is less effective. Aberration correction usually considers radial aberrations and ignores the effect of tangential aberrations, which can describe the nonlinear aberrations of the lens.

**Figure 13:**
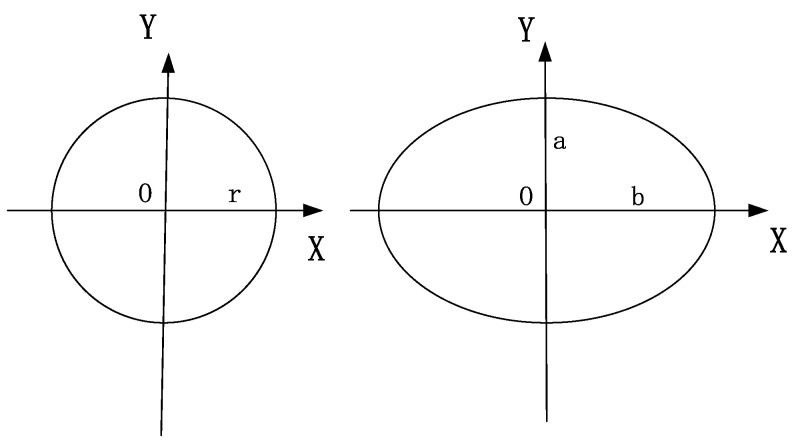
Standard-concentric-aberration model and elliptical-aberration-correction model.

Because the resolution ratio of the image H: V ≠ 1:1, there is a poor edge effect for the image edge distortion correction edge when using the standard concentric circle distortion model. Therefore, an elliptical distortion correction model is proposed using the improved standard concentric circle distortion model:

(18) $$x=x_{d}\frac{1}{\left(1+k_{1}r^{2}\right)},y=y_{d}\frac{1}{1+k_{1}r^{2}}$$

(19) $$\frac{x^{2}}{a^{2}}+\frac{y^{2}}{b^{2}}=r^{2}$$

where H is the height of the image, V represents the image’s width, a=H/2 and b=V/2 are shown in Figure 13.

The elliptical distortion correction model cannot cover the image completely ( H×V ). We further improve the elliptical distortion correction model using an approximate elliptical distortion correction model:

(20) $$x=k_{2}\frac{x_{d}}{1+k_{1}{\left(r-R\right)}^{2}},y=k_{2}\frac{y_{d}}{1+k_{1}{\left(r-R\right)}^{2}}$$

(21) $$\frac{x^{2}}{a^{2}}+\frac{y^{2}}{b^{2}}=r^{2}$$

where $k_{1}$ and $k_{2}$ represent the distortion coefficients; x and $x_{d}$ represent the corrected image and distorted image in the X-direction; and y and $y_{d}$ represent the corrected image and the distorted image in the Y-direction, respectively.

**Figure 14:**
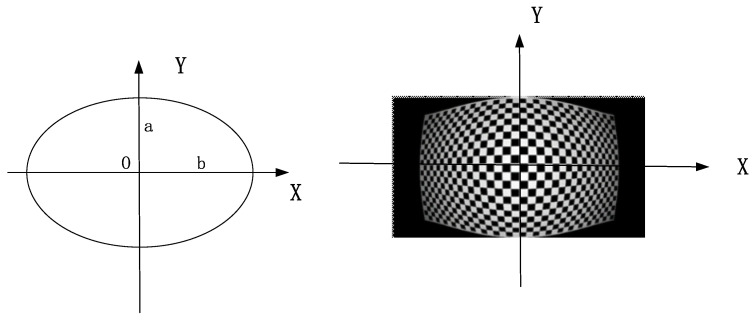
Correction-mapping table for approximate elliptic-distortion correction.

In this paper, real-time corrections are performed based on FPGA. A common approach in FPGA hardware circuits is the correction-mapping table, which first calculates the corresponding correction result of the image and stores it in the hardware circuit and determines the corresponding corrected video output from the correction-mapping table based on the input real-time video. The correction-mapping table is a straightforward computation but suffers from the disadvantage of depositing an approximate elliptic-distortion-correction-mapping table, which requires a large amount of hardware-circuit resources. Thus, we proposed elliptical distortion correction model only needs to store one quarter of the data in the hardware circuitry, as shown in Figure 14.

**Figure 15:**
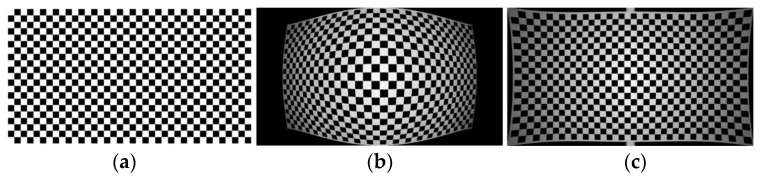
The 1920 × 1080 Visible light distortion effect: (**a**) standard checkerboard grid; (**b**) optical-system-aberration map (aberration rate −45%); and (**c**) aberration-correction-result map.

**Figure 16:**
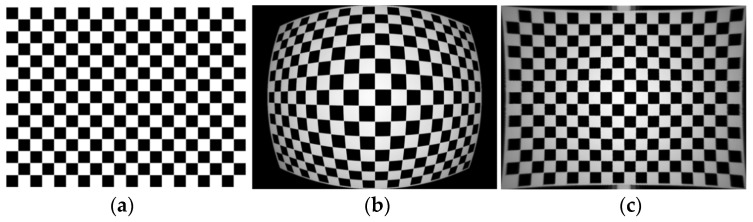
The 1024 × 768 infrared-distortion effect: (**a**) standard checkerboard grid; (**b**) optical-system-aberration map (aberration rate −45%); and (**c**) aberration-correction-result map.

The generated checkerboard grid H×V images are shown in Figure 15a and Figure 16a and are standard checkerboard grids of 1920 × 1080 and 1024 × 768, respectively. Figure 15b and Figure 16b present, respectively, the aberration simulation images derived using the standard checkerboard grid as the input image through the visible optical system model and those generated using the infrared optical system. Through the myopic elliptic-aberration model proposed in this paper for aberration correction, the aberration-correction map of the visible image and the aberration-correction map of the infrared image are obtained, as shown in Figure 15c and Figure 16c, respectively. The edges of the image are still aberrated, but the field-of-view is increased.

### 2.3. Infrared- and Visible Light-Fusion Algorithm

The infrared- and visible light images are horizontally aligned after image alignment and limit correction to realize the left and right images, and the common adaptive image-enhancement method [31] is used to enhance the infrared- and visible light images to improve the bright and dark regions in the image to improve the contrast of the image. Infrared- and visible light-image matching is a type of heterogenous spit matching, and it is difficult to find the same type of corresponding feature points for the two types of images. In this paper, we use the SURF + RANSAC algorithms to realize stereo matching [32,33]. The SURF algorithm has the characteristics of rotation, scale transformation, and brightness invariance; compared to the SIFT algorithm, SURF reduces the complexity of the algorithm, reduces the dimensionality of the feature descriptor from 128 to 65 dimensions, and reduces the computational amount by double [34]. The anomalous data in the matching process is filtered out by using the RANSAC algorithm.

Image fusion involves the use of image information from multiple imaging sensors in a unified scene to increase the perception of the scene and the ability to recognize targets and other objectives. Unlike traditional multi-scale fusion methods, this study adopts a multi-scale infrared- and visible light-fusion method based on the work in [35], which has the unique characteristics of retaining scale-specific information and reducing the edge halo, taking into account the different characteristics of the infrared image and the visible image. This study also adopts the traditional “maximum–absolute” fusion rule. With this optimization, the useful visual details can be better transferred to the fused image while suppressing the noise in the infrared image:

(22) $$V(p)=\left|I_{p}-I_{1}\right|+\left|I_{p}-I_{2}\right|+\cdot \cdot \cdot +\left|I_{p}-I_{N}\right|$$

where $I_{p}$ is the intensity value at pixel p in image I , V(p) is the significant value of pixel p , and N is the total number of pixels in image I .

If the two-pixel intensity values are equal, then

(23) $$V(p)=\sum_{j=0}^{L-1}M_{j}\left|I_{P}-I_{j}\right|$$

where j is the pixel point, $M_{j}$ is the number of pixels with pixel intensity the same as that of point j , and L is the number of gray levels in the image. The infrared image $I_{ir}$ and visible image $I_{vi}$ are the input images. Then, the base layer $B_{F}$ of the fused image is obtained as

(24) $$B_{F}=W_{b}B_{1}+\left(1-W_{b}\right)B_{2}$$

(25) $$W_{b}=0.5+\frac{I_{\mathrm{ir}}-I_{vi}}{2}$$

Infrared images usually contain coarse-scale structural information and noise and lack visual details compared with visible light images. The fused detail layer obtained by the “max–absolute” rule is improved to make the image look more natural and more suitable for human visual perception. The weighting factor $W^{j}$ of “max–absolute” is

(26) $$W^{j}=\{\begin{array}{c} 1,\left|d_{1}^{j}\right|>\left|d_{2}^{j}\right|(j=1,2,\cdot \cdot \cdot N)\\ 0,otherwise \end{array}.$$

Next, we apply a Gaussian filtering for denoising:

(27) $$W_{d}^{j}=Gaussian(W^{j},\delta_{s})$$

where setting $\delta_{s}=2$ , $M^{j}$ for the fusion details in the *j*_th_ layer is accomplished according to the “max–absolute” rule, as follows:

(28) $$M^{j}=W_{d}^{j}d_{1}^{j}+(1-W_{d}^{j})d_{2}^{j}.$$

The fusion detail layer $D^{j}$ of the *j*_th_ layer can be obtained according to the weighted least squares method:

(29) $$D^{j}=\sum_{P}\left({\left(D_{p}^{j}-M_{P}^{j}\right)}^{2}+\lambda a_{p}^{j}{\left(D_{p}^{j}-{\left(d_{2}^{j}\right)}_{p}\right)}^{2}\right)$$

where $a_{p}^{j}={\left(\left|\sum_{q\in w_{p}}{\left(d_{1}^{j}\right)}_{q}\right|+\varepsilon \right)}^{-1}$ represents the spatial variation weight; p represents the spatial position of the pixel; ε is a constant equal to 0.0001; and $w_{p}$ represents a rectangular window centered on pixel point p .

The fused image $I_{F}$ after combining the base layer $B_{F}$ and the detail layer $D^{1},D^{2},\ldots ,D^{N}$ is

(30) $$I_{F}=B_{F}+D^{1}+D^{2}+\cdot \cdot \cdot +D^{N}.$$

### 2.4. Binocular Stereo Ranging Algorithm

**Figure 17:**
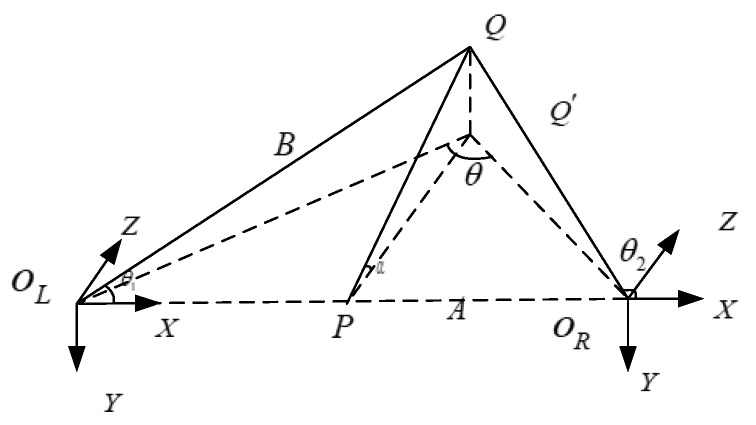
Schematic diagram of binocular stereo-ranging information.

Unlike the traditional binocular stereo-ranging method [36], we seek to acquire binocular stereo-ranging information from the image obtained via aberration correction and image fusion. As shown in Figure 17, parallax information is used to restore the depth. Here, $Q_{L}$ and $Q_{R}$ are the coordinate origins of the two camera coordinate systems, and A is the baseline length. To find the corresponding point of the world coordinate system in the stereoimage pair for point Q , the corresponding direction vectors in the virtual coordinate system are $\vec{Q_{L}Q}$ , $\vec{Q_{R}Q}$ . $Q^{\prime }$ is the projection of Q in the $XO_{L}Z$ plane. The angles of the vector $\vec{O_{L}Q}$ , $\vec{O_{R}Q}$ with the plane $YO_{L}Z$ , $YO_{R}Z$ are $\beta_{1}$ , $\beta_{2}$ . If the angles with the $O_{L}Z$ -axis and $O_{2}Z$ -axis are both α ; then, we apply the following:

(31) $$\theta_{1}=\frac{\pi }{2}-\beta_{1}\;,\;\theta_{2}=\frac{\pi }{2}-\beta_{2}.$$

(32) $$\frac{B}{\sin(\pi -\theta_{2})}=\frac{A}{\sin\theta }=\frac{A}{\sin(\theta_{2}-\theta_{1})}\Rightarrow B=\frac{A\sin\theta_{2}}{\sin(\theta_{2}-\theta_{1})}.$$

Then, the depth information of Q is

(33) $$Qdepth=Q^{\prime }P=B\sin\theta_{1}\cos a.$$

## 3. Experiments and Results

### 3.1. Test Platform

**Figure 18:**
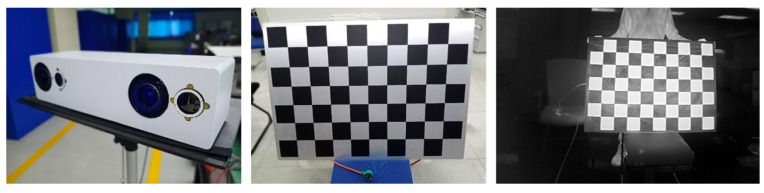
Wide-field-of-view binocular stereo sensing system and calibrated checkerboard grid.

The experiment adopted a wide-field-of-view binocular stereo perception system and a calibration checkerboard grid (ambient and heated state), as shown in Figure 18. The calibration checkerboard grid was based on the Boltier principle design for an active infrared-radiation-calibration checkerboard grid. We used a JY-260 microcomputer temperature controller device from Jiangyin Jinyu Electric Heating Appliance Co., Ltd. (Jiangsu, China) to control the temperature of the calibration checkerboard grid in a range from −50 °C to −260 °C, the calibration checkerboard grid was 10 × 7, the length of the squares was 100 mm, and the overall size was 1040 mm × 740 mm. Infrared-component calibration was carried out using the power supply. After the infrared component was calibrated, the power supply was used to heat the calibration checkerboard grid, and the temperature was controlled with the JY-260 microcomputer temperature controller at 50 °C, which heated and cooled the white box to produce corners with a large grayscale gradient on the infrared image, which was convenient for corner identification.

### 3.2. Calibration Test Results

The binocular stereo sensing system was calibrated using sensors for tessellated grids in a three-dimensional scene. Under the optical imaging model, the relationship between the spatial coordinate system of the object points in the scene and the corresponding image points in the image plane was established, which, in turn, determined the internal and external parameters of the camera. In the experiment, calibration of the internal and external parameters of infrared and visible cameras for binocular stereo sensing systems was based on Taylor model-calibration. The images used in this experiment were acquired by visible light and infrared left and right cameras in different orientations for calibrating the checkerboard grid, and then the coordinates of the corner points were extracted.

**Figure 19:**
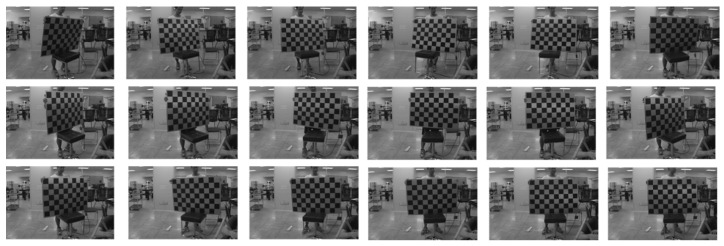
Calibration chart for the visible light camera (left lens).

**Figure 20:**
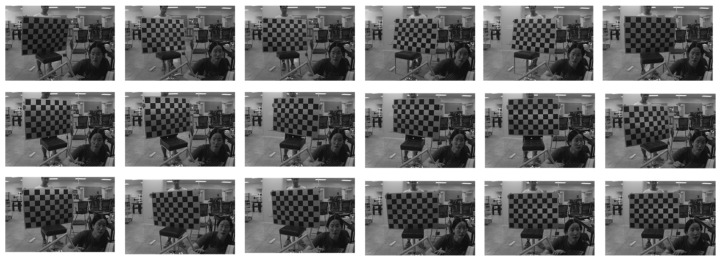
Calibration chart for the visible light camera (right lens).

**Figure 21:**
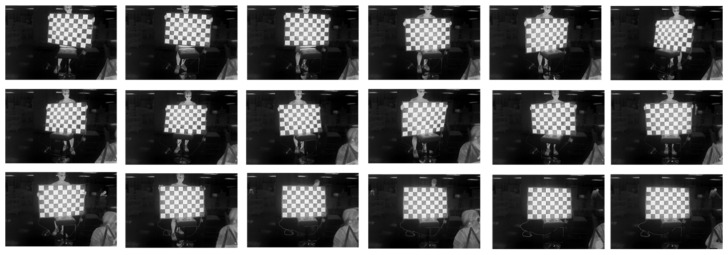
Calibration chart for the infrared camera (left lens).

**Figure 22:**
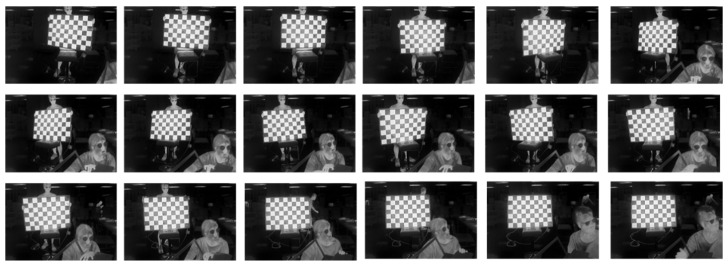
Calibration chart for the infrared camera (right lens).

We selected 18 images from the collected data for calibration, as shown in the binocular stereo left lens’s visible light-camera-calibration diagram in Figure 19, and in the right lens’s visible light camera-calibration diagram in Figure 20, and in the right lens’s infrared camera-calibration diagram in Figure 21, and in the right lens’s infrared camera-calibration diagram in Figure 22. The binocular stereo sensing system’s visible camera and its infrared camera inside and outside the parameters of the calibration results are shown in Table 4 and Table 5. We perform parameter calibration based on a cal ibrated chessboard, which absorbs heat uniformly after heating, overcoming the problem of difficult to recognize corner points in infrared cameras, and at the same time solving the calibration problems arising from the different imaging characteristics of infrared and visible light.

### 3.3. Aberration-Correction Results

**Figure 23:**
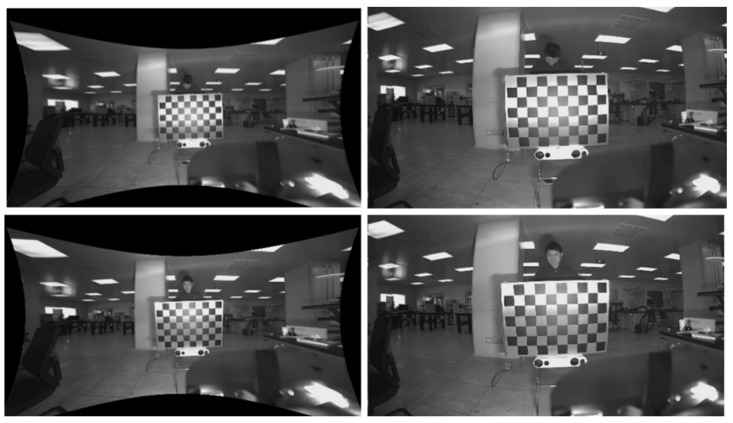
Aberration and correction maps for visible light camera (left lens).

**Figure 24:**
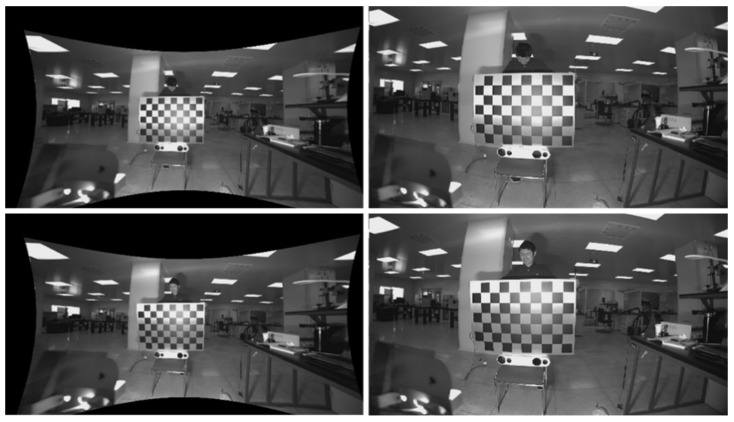
Aberration and correction maps for visible light camera (right lens).

The aberration maps for this experiment were acquired indoors on a calibrated checkerboard grid using a binocular stereo sensing system. The aberration-correction algorithm in this paper was used to obtain the aberration-correction map (shown in Figure 23 and Figure 24). The aberration rate of the visible image was 45%, indicating that the image aberration caused by the large field-of-view was greatly improved at the edges and that the field-of-view was not lost.

### 3.4. Ranging Test Results

**Figure 25:**
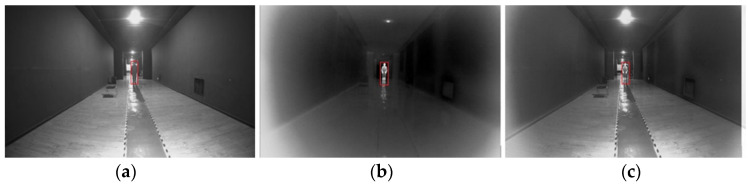
Binocular distance-measurement: (**a**) visible image; (**b**) infrared image; (**c**) and fused image.

**Figure 26:**
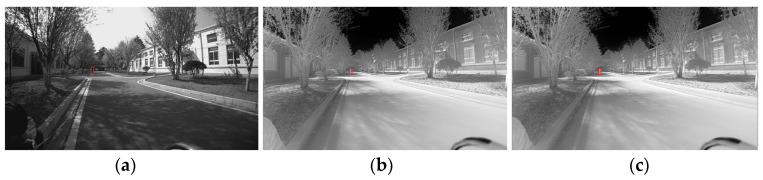
Binocular ranging-scene diagram in the park: (**a**) visible light camera image; (**b**) infrared image; (**c**) and fused image.

As shown in Figure 25 and Figure 26, a person was selected as the recognition target and could be recognized by both visible light and infrared cameras. Several ranging measurements were taken at 5 m, 10 m, 15 m, 20 m, 25 m, 30 m, 40 m, and 60 m. By recognizing the target, the target point was framed out and the depth value of the feature point inside the frame was calculated. Sometimes the feature point contained both the foreground and the background feature points. In this study, the analysis was carried out using Equation (34), eliminating the unwanted data. The results of the ranging tests are shown in Table 6.

(34) $$\bar{d_{V}}\pm \frac{\sum \left|d_{i}-\bar{d_{V}}\right|}{n}$$

where $d_{i}$ is the distance value of the feature point, and $\bar{d_{V}}$ is the average value of the distance value of the feature point.

Table 6 shows the visible ranging error at 0.99 m (distance 20 m), 1.62 m (distance 30 m), and 3.24 m (distance 60 m) and 0.02 m (distance 20 m), 0.47 m (distance 30 m), and 2.55 m (distance 60 m), as well as the fusion-ranging error at 0.16 m (distance 20 m), 0.37 m (distance 30 m), and 0.35 m (distance 60 m). At a close distance, the error of ranging was small, and the error gradually increased with an increase in distance. Comparing the ranging results for visible and infrared cameras, the ranging accuracy of infrared camera was better than that of visible light camera. The reason for this result is that the reprojection error for the calibration of the infrared camera was less than that of the calibration results for the visible light camera. Our proposed fusion-ranging method has greater accuracy than those using only infrared- or visible light. The reason for this result is that the fusion algorithm combines the advantages of visible- light and infrared, and the extracted feature points are more accurate, so its ranging errors were 0.16 m (distance 20 m), 0.37 m (distance 30 m), and 0.35 m (distance 60 m). In addition, its feature points were more accurate, so its ranging results were higher.

**Figure 27:**
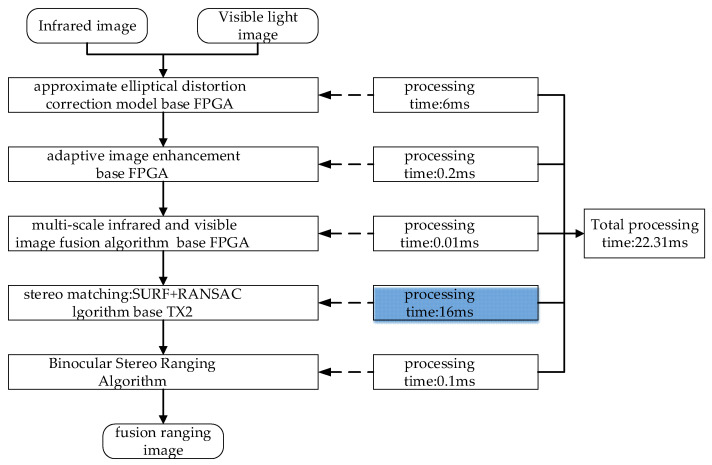
Image-algorithm-processing time for binocular stereo sensing systems.

In order to improve the performance of this system, this paper improves the running speed of the algorithms by optimizing the related algorithms, parallel processing, and hardware acceleration, as shown in Figure 27. The major algorithms include: an FPGA-based elliptic-aberration correction, which saves FPGA storage space by storing 1/4 of the data, and the processing time of the algorithm is 6 ms; an FPGA-based adaptive image-enhancement algorithm with a processing time of 0.2 ms, lower computational complexity, and faster processing time; FPGA-based multi-scale infrared and visible fusion with a processing time of 0.01 ms; a TX2-based SURF + RANSAC binocular stereo-matching algorithm which uses feature point extraction, feature point descriptor calculation, matching point search and consistency checking, such that the computational complexity is high and the processing time is 16 ms; and, finally, a binocular stereo ranging method that removes the misjudged points to get the final ranging data with a processing time of about 0.1 ms. To summarize, the algorithms in the binocular stereo sensing system have a total processing time of about 22.31 ms.

## 4. Conclusions

This study proposed a dual-band fusion binocular stereo perception system with a large 120° field-of-view combining infrared- and visible light imaging through two sets of binocular stereo vision. This system enables information acquisition and stereo visual perception within a wide field-of-view, providing enhanced scene understanding and addressing the limitations of traditional binocular stereo vision systems, such as a narrow field-of-view and inaccuracies in short-distance ranging. Aiming to solve the aberration problems of infrared and visible images with a large field-of-view, this paper adopted the elliptic-aberration-correction model, which was corrected in real-time in the hardware circuit in FPGA, thereby saving the storage resources of FPGA and solving the problem of field-of-view loss. By adopting the multi-scale fusion method, the visible and infrared images compensated for each other’s differences, fully utilized the texture information of the visible image and the thermal radiation contour information of the infrared image, and significantly improved the ranging accuracy. The simulation and experimental results demonstrated that the fusion-ranging accuracy outperforms the individual ranging accuracies for infrared- and visible light cameras.

The proposed system can maintain high performance under different light and weather conditions and scenarios. Adding image recognition algorithms for target localization of special targets or obstacles, which has application value in many areas such as driverless vehicles, virtual reality, and robot navigation. With the advancement of hardware and algorithm technology, future research will focus on enhancing the real-time performance of binocular stereo perception algorithms and achieving low-power edge computing, engineering the binocular stereo perception system, and further building the binocular stereo perception system with different resolutions to correct aberrations and accurately recognize objects at different distances. By introducing artificial intelligence algorithms, the system can realize adaptive processing and intelligent decision-making for complex scenes.

## Figures and Tables

**Table 1:** Optical-design specifications for the visible light lens.

Parameters	Value	Parameters	Value
Objective focal length (f)	10.96 mm	Diagonal field	133°
F-number	2.1	Distortion (maximum on the diagonal)	−45%
Horizontal field	124°	Maximum working distance	97.16 m
Vertical field	74.4°		

**Table 2:** The optical-design specifications for the infrared objective lens.

Parameters	Value
objective focal length (f)	6 mm
F-number	2.1
horizontal field	121.6°
vertical field	107°
distortion (maximum on the diagonal)	−47%

**Table 3:** Infrared- and visible light-ranging accuracy.

Serial Number	Measuring Distance (L)	Visible Light-Ranging Accuracy (L)	Infrared-Ranging Accuracy (L)
1	15 m	dL≥7.63cm	dL≥15.00cm
2	30 m	dL≥30.50cm	dL≥60.00cm

**Table 4:** Internal and external parameters of the visible light camera of the binocular stereo sensing system.

Parameter	Calibration Results (Left)	Calibration Results (Right)
(c,d,e)	$\left(1.0001,-1.2\times 10^{-5},1.7\times 10^{-5}\right)$	$\left(1.0001,-2.2\times 10^{-5},-2.12\times 10^{-4}\right)$
$\left(u_{0},v_{0}\right)$	(487.0978,964.3319)	(464.1441,949.0153)
$\left(a_{0},a_{1},a_{2},a_{3},a_{4}\right)$	$\begin{array}{l} (-835.384,0,3.296\times 10^{-4},\\ 3.290\times 10^{-7},-3.189\times 10^{-10}) \end{array}$	$\begin{array}{l} (-819.290,0,3.642\times 10^{-4},\\ 2.727\times 10^{-7},-2.909\times 10^{-10}) \end{array}$
R	$\left(\begin{array}{ccc} 0.9976 & -0.0009 & 0.0684\\ 0.0007 & \;0.9999 & 0.0019\\ -0.0684 & -0.0018 & 0.9976 \end{array}\right)$	
T	$\left[\begin{array}{ccc} -335.5285 & 9.9994 & -16.8602 \end{array}\right]$	

**Table 5:** Internal and external parameters for the infrared camera of the binocular stereo sensing system.

Parameter	Calibration Results (Left)	Calibration Results (Right)
(c,d,e)	$\left(0.992,-1.47\times 10^{-3},6.2\times 10^{-5}\right)$	$\left(0.9998,-9.5\times 10^{-5}\;,1.9\times 10^{-5}\right)$
$\left(u_{0},v_{0}\right)$	(479.4945,959.2869)	(449.2642,932.4781)
$\left(a_{0},a_{1},a_{2},a_{3},a_{4}\right)$	$\begin{array}{l} (-780.385,0,2.472\times 10^{-3},\\ 1.522\times 10^{-8},-7.214\times 10^{-12}) \end{array}$	$\begin{array}{l} (-796.383,0,8.938\times 10^{-5},\\ 3.160\times 10^{-8},1.7333\times 10^{-10}) \end{array}$
R	$\left(\begin{array}{ccc} 0.9989 & 0.0371 & 0.0291\\ -0.0375 & \;0.9992 & 0.0133\\ -0.0286 & -0.0144 & 0.9995 \end{array}\right)$	
T	$\left[\begin{array}{ccc} -355.90 & 11.0089 & 35.05714 \end{array}\right]$	

**Table 6:** Binocular distance-measurement results.

Serial Number	Measuring Distance (m)	Visible Light-Ranging		Infrared Ranging		Fusion-Ranging	
		Distance(m)	Difference (m)	Distance (m)	Difference (m)	Distance (m)	Difference (m)
1	5	4.82	0.18	5.05	0.05	5.01	0.01
2	10	10.15	0.15	10.02	0.02	10	0
3	15	14.64	0.36	15.04	0.04	15.02	0.02
4	20	19.01	0.99	19.84	0.16	19.98	0.02
5	25	24.14	0.86	24.53	0.47	24.86	0.14
6	30	31.62	1.62	29.53	0.47	30.37	0.37
7	40	37.99	2.01	39.46	0.54	39.78	0.22
8	60	56.76	3.24	62.55	2.55	60.35	0.35

## Data Availability

Data are contained within the article.
